# Na_1.67_Mn_2.17_(MoO_4_)_3_

**DOI:** 10.1107/S160053681400614X

**Published:** 2014-03-26

**Authors:** Chahira Bouzidi, Mohamed Faouzi Zid, Ahmed Driss, Wafa Frigui

**Affiliations:** a Laboratoire de Matériaux et Cristallochimie, Faculté des Sciences de Tunis, Université de Tunis ElManar, 2092 Manar II Tunis, Tunisia

## Abstract

The title compound, disodium dimanganese(II) tris­[ortho­molybdate(VI)], was prepared by solid-state reactions. The structure can be described as being composed of Mn_2_Mo_2_O_14_ double-chains that are inter­connected by corner-sharing with MoO_4_ tetra­hedra, leading to a three-dimensional framework with channels propagating in [100] and [001] in which the Na^+^ counter-cations are located. One of these Na sites is located on an inversion centre, one is partially occupied [occupancy 0.341 (9)], and one is statistically occupied by Na and Mn in a ratio of 0.829 (5):0.171 (5). Na_1.67_Mn_2.17_(MoO_4_)_3_ is isotypic with structures of the Ag_2_ *M*_2_(MoO_4_)_3_ (*M* = Zn, Mg, Co, Mn) family. A comparative structural description is provided between the structure of the title compound and those of related phases containing (*MX*O_8_)_*n*_ chains (*M* = Mo, Mn and *X* = As) or *M*_2_O_10_ (*M* = Mo, Mn, Nb, V) dimers.

## Related literature   

For isotypic compounds, see: Tsyrenova *et al.* (2004 ▶); Gicquel-Mayer *et al.* (1981 ▶); Balsanova *et al.* (2009 ▶). For background to the physico-chemical properties of related compounds, see: Gueho *et al.* (1993 ▶); Piffard *et al.* (1985 ▶); Solodovnikov *et al.* (1986 ▶, 1997 ▶, 1998 ▶); Prabaharan *et al.* (1997 ▶). For details of structurally related compounds, see: Frigui *et al.* (2011 ▶, 2012 ▶); Guesmi & Driss (2012 ▶); Belkhiri *et al.* (2009 ▶); Chérif *et al.* (2011 ▶); Ledain *et al.* (1996 ▶); Ezzine Yahmed *et al.* (2013 ▶). For bond lengths and bond-valence sums, see: Souilem *et al.* (2014 ▶); Ennajeh *et al.* (2013 ▶); Brown & Altermatt (1985 ▶).

## Experimental   

### 

#### Crystal data  

Na_1.67_Mn_2.17_(MoO_4_)_3_
*M* *_r_* = 637.43
Triclinic, 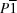

*a* = 7.1072 (7) Å
*b* = 8.8120 (8) Å
*c* = 10.4330 (9) Å
α = 106.501 (9)°
β = 105.361 (9)°
γ = 102.756 (8)°
*V* = 572.31 (11) Å^3^
*Z* = 2
Mo *K*α radiation
μ = 5.64 mm^−1^
*T* = 298 K
0.28 × 0.15 × 0.11 mm

#### Data collection  

Enraf–Nonius CAD-4 diffractometer
Absorption correction: ψ scan (North *et al.*, 1968 ▶) *T*_min_ = 0.371, *T*_max_ = 0.540
3079 measured reflections
2474 independent reflections
2206 reflections with *I* > 2σ(*I*)
*R*_int_ = 0.017
2 standard reflections every 120 min intensity decay: 1.3%

#### Refinement  

*R*[*F* ^2^ > 2σ(*F* ^2^)] = 0.030
*wR*(*F* ^2^) = 0.083
*S* = 1.09
2474 reflections
180 parameters
Δρ_max_ = 1.52 e Å^−3^
Δρ_min_ = −1.24 e Å^−3^

### 

Data collection: *CAD-4 EXPRESS* (Duisenberg, 1992 ▶; Macíček & Yordanov, 1992 ▶); cell refinement: *CAD-4 EXPRESS*; data reduction: *XCAD4* (Harms & Wocadlo, 1995 ▶); program(s) used to solve structure: *SHELXS97* (Sheldrick, 2008 ▶); program(s) used to refine structure: *SHELXL97* (Sheldrick, 2008 ▶); molecular graphics: *DIAMOND* (Brandenburg & Putz, 2001 ▶); software used to prepare material for publication: *WinGX* (Farrugia, 2012 ▶).

## Supplementary Material

Crystal structure: contains datablock(s) I. DOI: 10.1107/S160053681400614X/wm5009sup1.cif

Structure factors: contains datablock(s) I. DOI: 10.1107/S160053681400614X/wm5009Isup2.hkl

CCDC reference: 992636

Additional supporting information:  crystallographic information; 3D view; checkCIF report

## Figures and Tables

**Table 1:** Selected bond lengths (Å)

Mo1—O2	1.726 (4)
Mo1—O6^i^	1.772 (4)
Mo1—O10	1.787 (4)
Mo1—O9	1.812 (4)
Mo2—O5^ii^	1.739 (4)
Mo2—O11	1.777 (4)
Mo2—O4	1.778 (4)
Mo2—O7	1.795 (4)
Mo3—O1	1.742 (5)
Mo3—O3	1.747 (4)
Mo3—O12	1.757 (5)
Mo3—O8	1.790 (4)
Mn1—O12^iii^	2.060 (4)
Mn1—O6	2.148 (4)
Mn1—O9	2.193 (4)
Mn1—O9^iii^	2.224 (4)
Mn1—O4	2.224 (4)
Mn1—O11^iii^	2.226 (4)
Mn2—O3^iv^	2.115 (4)
Mn2—O5	2.151 (4)
Mn2—O7^v^	2.153 (4)
Mn2—O10^iii^	2.161 (4)
Mn2—O8	2.194 (4)
Mn2—O10^vi^	2.264 (4)
Na3—O11^iii^	2.212 (4)
Na3—O1^vii^	2.250 (7)
Na3—O8^viii^	2.250 (4)
Na3—O7^ix^	2.279 (4)
Na1—O2^iv^	2.376 (4)
Na1—O2^x^	2.376 (4)
Na1—O4^xi^	2.430 (4)
Na1—O4	2.430 (4)
Na1—O1^iii^	2.797 (6)
Na1—O1^xii^	2.797 (6)
Na2—O2^xiii^	2.408 (11)
Na2—O2^iii^	2.529 (12)
Na2—O4^iii^	2.815 (13)
Na2—O1	2.990 (12)

Symmetry codes: (i) 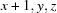; (ii) 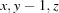; (iii) 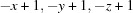; (iv) 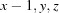; (v) 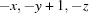; (vi) 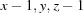; (vii) 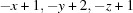; (viii) 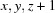; (ix) 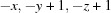; (x) 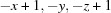; (xi) 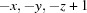; (xii) 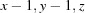; (xiii) 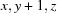.

**Fig. 1.**
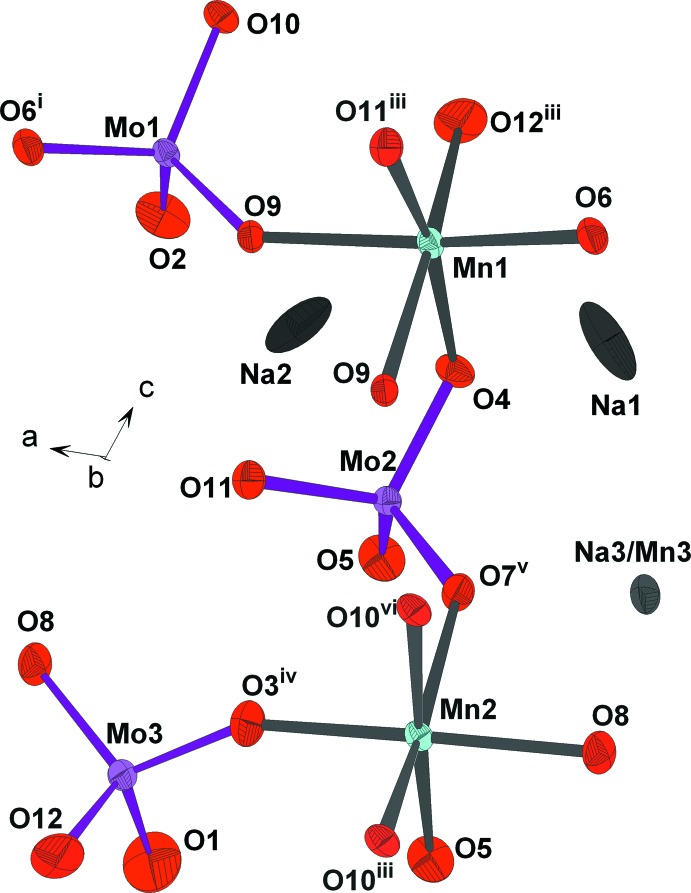
Unité asymétrique dans Na1,67Mn2,17(MoO4)3. Les éllipsoïdes ont été définis avec 50% de probabilité. [Code de symétrie: (i) x - 1, y - 1, z; (ii) x, y - 1, z; (iii) -x + 1, -y + 1, -z + 1; (iv)x - 1, y, z; (v) -x + 1, -y, -z + 1; (vi) x + 1, y, z; (vii) -x + 2, -y + 1, -z + 1.]

**Fig. 2.**
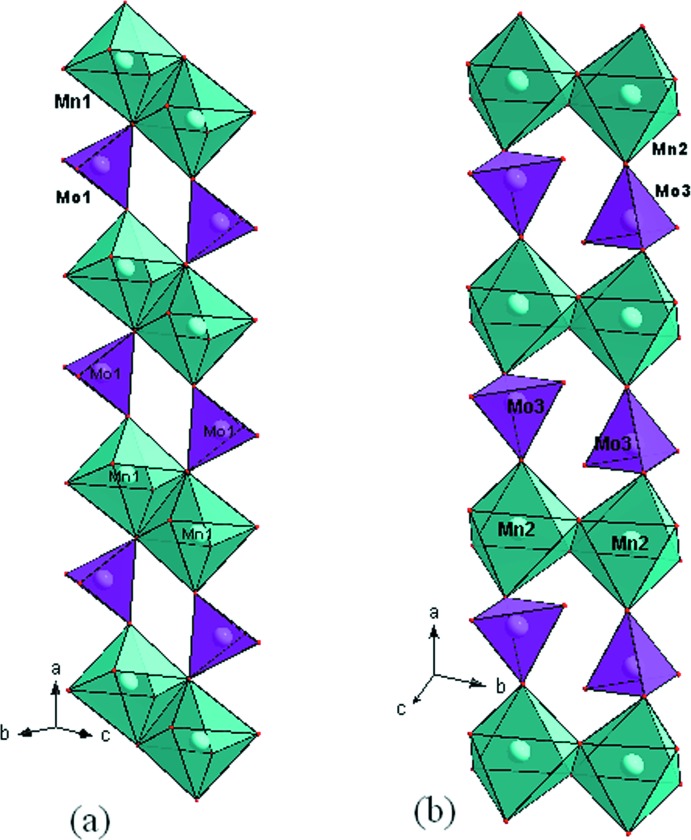
Représentation des chaînes doubles: (a) Mn(2)2Mo(3)2O14 (b) Mn(1)2Mo(2)2O14.

**Fig. 3.**
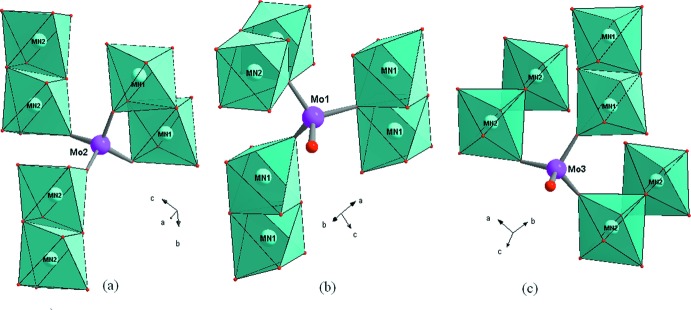
Représentation des environnements des tétraèdres: (a) Mo(2)O4 (b) Mo(1)O4 (c) Mo(3)O4.

**Fig. 4.**
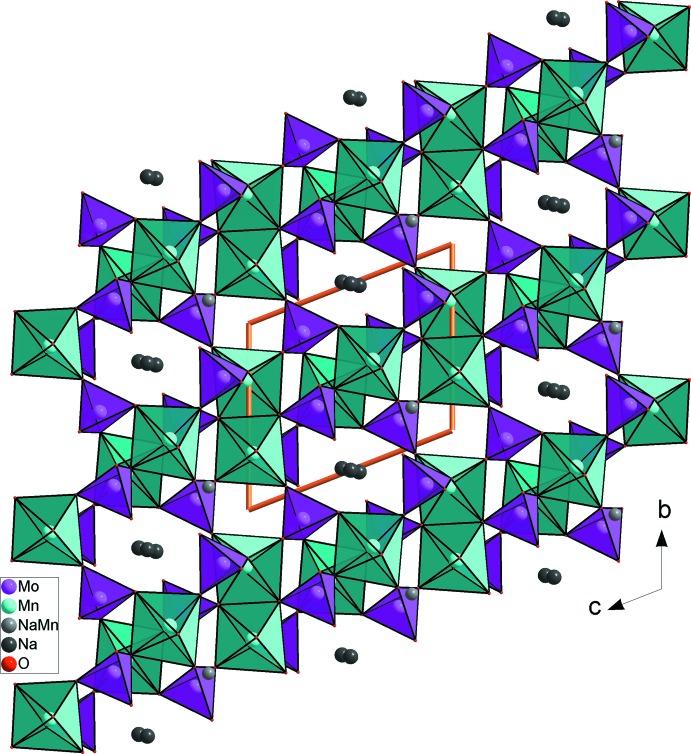
Projection de la structure de Na1,67Mn2,17(MoO4)3 selon [100].

**Fig. 5.**
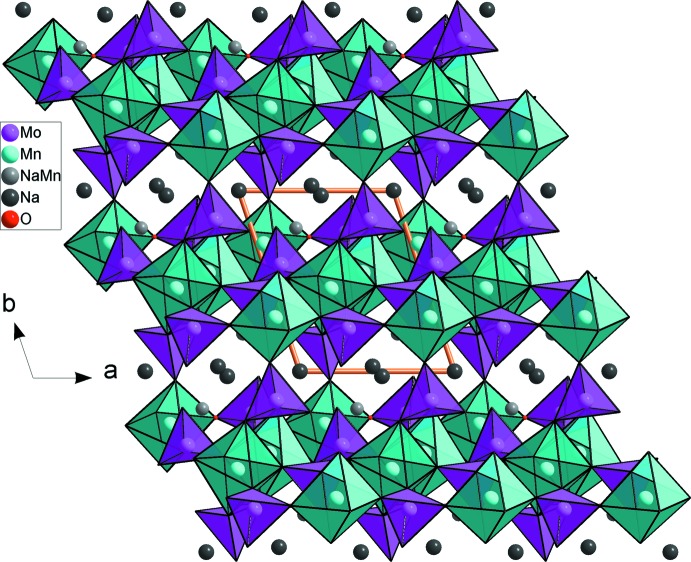
Projection de la structure de Na1,67Mn2,17(MoO4)3 selon [001].

## supplementary crystallographic information

### 1. Comment

L'élaboration des matériaux à charpente ouvertes formées d'octaèdres et de tétraèdres a connue un essor considérable. En effet, ils ont fait l'objet de très nombreux travaux de recherche au niveau international. Dans ce contexte, les matériaux inorganiques connaissent une forte expansion en raison notamment de leurs propriétés physico-chimiques performentes en relation directe avec leurs structures cristallines ainsi que leurs applications notamment: conduction ionique (Gueho *et al.*, 1993; Prabaharan *et al.*, 1997) et échange d'ions (Piffard *et al.*, 1985). Dans ce cadre, et en vue de synthétiser de nouveaux matériaux à charpente ouvertes, nous avons exploré les systèmes *A*–Mn–Mo–O (*A* = ion monovalent) dans lesquels différentes phases ont été précédemment isolées: K_2_Mn_2_Mo_3_O_12_ (Solodovnikov *et al.*, 1986), K_10_MnMo_7_O_27_, (Solodovnikov *et al.*, 1997), K_4_MnMo_4_O_15_ (Solodovnikov *et al.*, 1998). Nos tentatives de synthèse nous ont permis de trouver une nouvelle phase de formulation Na_1,67_Mn_2,17_(MoO_4_)_3_ qui a été élaborée par réaction à l'état solide à 998 K.

L'unité asymétrique dans Na_1,67_Mn_2,17_(MoO_4_)_3_ est construite par deux octaèdres MnO_6_ et trois tétraèdres MoO_4_ liés par mise en commun de sommets. La compensation de charges est assurée par les cations Na^+^ (Fig 1). La structure peut être décrite au moyen de chaînes classiques de type Mn(2)Mo(3)O_8_ reliées par mise en commun d'arrêtes entre octaèdres pour former des doubles chaînes Mn(2)_2_Mo(3)_2_O_14_ disposées selon la direction [100] (Fig. 2*a*). D'autre part, les polyèdres Mn(1)O_6_ et les tétraèdres Mo(1)O_4_ se connectent pour conduire à des chaînes Mn(1)_2_Mo(1)_2_O_14_ dans lesquelles les tétraèdres Mo(1)O_4_ se lient pour former des ponts triples 2Mn(1)=O–Mo(1) avec les dimères Mn(1)_2_O_10_ (Fig. 2*b*). Une disposition particulière de groupements Mn(1)_2_O_10_ et Mn(2)_2_O_10_ permet, par partage de sommets entre polyèdres de nature différente, la connexion des doubles chaînes Mn(1)_2_Mo(1)_2_O_14_ et Mn(2)_2_Mo(3)_2_O_14_. En effet, chaque tétraèdre Mo(2)O_4_ partage ses quatre sommets avec seulement trois dimères (Fig. 3*a*). Par contre un tétraèdre Mo(1)O_4_ lie d'une part deux dimères différents par formation de deux ponts triples de type 2Mn(1)=O–Mo(1) et d'autre part, il se connecte, par pont simple, à un groupement Mn(1)_2_O_10_ (Fig. 3 b). Les tétraèdres Mo(3)O_4_ renforcent d'avantage, la jonction des chaînes par formation de ponts mixtes de type Mn(1)–O–Mo(3) et Mn(2)–O–Mo(3) (Fig. 3*c*). Il est à signaler que le quatrième sommet, restant libre, dans chaque tétraèdre Mo(1)O_4_ et Mo(3)O_4_, forme un groupement molybdyl (Mo–O_L_). Il en résulte donc, une charpente tridimensionnelle possédant des canaux où logent les cations Na^+^ (Fig. 4 et 5). Dans chacun de ces tétraèdres, on relève des distances moyennes, d(Mo–O) de l'ordre de 1,768 (2) Å, conforment à celles observées dans la littérature (Souilem *et al.*, 2014; Ennajeh *et al.*, 2013). Concernant les tétraèdres Mo(1)O_4_ et Mo(3)O_4_ on relève une distance longue pour l'oxygène engagé dans le pont mixte et une plus courte qui correspond aux atomes d'oxygène non reliés par ailleurs (Fig. 3*b* et 3*c*). De plus, les distances moyennes Mn–O, et Na–O, égales respectivement à 2,176 (2) Å et 2,492 (2) Å, sont comparables à celles rencontrées dans des travaux antérieurs (Frigui *et al.*, 2012; Guesmi & Driss, 2012). Ainsi, le calcul des différentes valences de liaison (BVS), utilisant la formule empirique de Brown (Brown & Altermatt, 1985), conduit aux valeurs des charges des ions suivants: Mo1 (5,81), Mo2 (5,83), Mo3 (5,94), Mn1 (2,01), Mn2 (2,02), Na1(0,93), Na2(0,61), Na3/Mn3 (1,28), ce qui confirme les degrés d'oxydation des différents ions attendus dans la structure. Un examen bibliographie montre que la phase caractérisée est isostructurale à celles de formulation Ag_2_*M*_2_(MoO_4_)_3_ (*M* = Mg, Co, Mn) (Tsyrenova *et al.*, 2004) (*M* = Zn, Fe) (Gicquel-Mayer *et al.*, 1981; Balsanova *et al.*, 2009). Elle est construite à partir de chaînes MnMoO_8_. Ce type de chaînes est observé dans d'autres composés rencontrés dans la littérature notamment: K_1,65_V_1,78_W_0,22_O_2_(AsO_4_)_2_ (Belkhiri *et al.* 2009) et Ag_1,09_Mn_3.46_(AsO_4_)_3_ (Frigui *et al.* 2012). En effet, dans l'arséniate K_1,65_V_1,78_W_0,22_O_2_(AsO_4_)_2_ les octaèdres *M*O_6_ (*M* = W/V ou V) et les tétraèdres AsO_4_ sont reliés par mise en commun de sommets pour former deux chaînes similaires [*M*AsO_8_] et [VAsO_8_] disposées respectivement selon *a* et *c*. Leur connexion est assurée seulement par formation de ponts mixtes entre octaèdres et tétraèdres. Contrairement à notre structure où les chaînes [MnMoO_8_] se connectent par mise en commun d'arêtes (Fig. 2*a* et 2*b*). Une comparaison avec celle du triarséniate Na_0.5_K_0.65_Mn_3.43_(AsO_4_)_3_ (Frigui *et al.*, 2011) montre une différence nette dans la charpente et en particulier dans les types de connexion des groupements Mn_2_O_10_ mis en jeu. En effet, on remarque que dans le cas de Na_0.5_K_0.65_Mn_3.43_(AsO_4_)_3_, les dimères Mn_2_O_10_ sont liés entre eux par partage d'arêtes pour conduire à des chaînes infinies d'octaèdes connectées avec les tétraèdres AsO_4_ par mise en commun des sommets. Dans notre cas, les dimères Mn_2_O_10_ restent isolés et se lient directement aux tétraèdres MoO_4_ par partage de sommets. De plus la recherche de structures présentant des aspects communs avec celle de Na_1,67_Mn_2,17_(MoO_4_)_3_ nous a conduit aux composés K_0.12_Na_0.54_Ag_0.34_Nb_4_O_9_AsO_4_ (Chérif *et al.*, 2011) et LiMo_2_O_3_(PO_4_)_3_ (Ledain *et al.*, 1996). En effet, dans K_0.12_Na_0.54_Ag_0.34_Nb_4_O_9_AsO_4_ les dimères Nb_2_O_10_ s'insèrent entre les couches et assurent leur connexion par mise en commun de sommets entre octaèdres. Dans LiMo_2_O_3_(PO_4_)_3_, les dimères Mo_2_O_11_ se lient au moyen de sommets pour conduire à une charpente tridimensionnelle. Par contre, dans Li[VMoO_6_] (Ezzine Yahmed *et al.*, 2013) les dimères VMoO_10_ se lient par partage d'arêtes pour former des chaînes doubles disposées en zigzag qui se connectent à leur tour par ponts simples donnant des couches en dents de scie, ce qui mène à une structure bidimensionnelle.

### 2. Experimental

Un mélange de Na_2_CO_3_ (Prolabo, 27778), C_9_H_9_MnO_6_·2H_2_O (Fluka, 63538) et (NH_4_)_2_Mo_4_O_13_ (Fluka, 69858) sont pris dans les proportions Na:Mn:Mo égales à 2:2:3. L'ensemble est finement broyé et mis dans un creuset en porcelaine. Il est préchauffé jusqu'à 623 K afin d'éliminer les produits volatils. Le résidu a été ensuite porté à 998 K (proche de la température de fusion) et maintenu à cette dernière pendant trois semaines pour favoriser la germination et la croissance des cristaux. Un refroidissement lent (5 K/24 h) a été appliqué jusquà 900 K suivi d'un autre plus rapide (50 K/jour) jusqu'à la température ambiante. Des cristaux de couleur jaunâtre ont été séparés par l'eau chaude.

### 3. Refinement

L'affinement de tous les paramètres variables conduit à des ellipsoïdes bien définis. Les densités d'électrons maximum et minimum restants dans la Fourier-différence sont situées respectivement à 0,92 Å de Mo3 et à 0,89 Å de Mo2. Il en résulte la composition chimique finale, Na_1,67 (1)_Mn_2,17 (1)_(MoO_4_)_3_ du nouveau matériau obtenu. Le cristal étant de petite taille la correction d'absoption par psi-scan n'a eu aucun effet sur le résultat de l'affinemet final sauf une légère augmentation des valeurs absolues des densités d'électrons maximum (de 0,81 à 1,47) et minimum (de -0,74 à -1,28) situées respectivement à 0,96 Å de Mn1 et à 0,89 Å de Mo1.

### Crystal data

**Table:**

Na_1.67_Mn_2.17_(MoO_4_)_3_	*Z* = 2
*M**_r_* = 637.43	*F*(000) = 589
Triclinic, *P*1	*D*_x_ = 3.699 Mg m^−^^3^
Hall symbol: -P 1	Mo *K*α radiation, λ = 0.71073 Å
*a* = 7.1072 (7) Å	Cell parameters from 25 reflections
*b* = 8.8120 (8) Å	θ = 10–15°
*c* = 10.4330 (9) Å	µ = 5.64 mm^−^^1^
α = 106.501 (9)°	*T* = 298 K
β = 105.361 (9)°	Prism, yellow
γ = 102.756 (8)°	0.28 × 0.15 × 0.11 mm
*V* = 572.31 (11) Å^3^	

### Data collection

**Table:**

Enraf–Nonius CAD-4 diffractometer	2206 reflections with *I* > 2σ(*I*)
Radiation source: fine-focus sealed tube	*R*_int_ = 0.017
Graphite monochromator	θ_max_ = 27.0°, θ_min_ = 2.2°
ω/2θ scans	*h* = −1→9
Absorption correction: ψ scan (North *et al.*, 1968)	*k* = −11→11
*T*_min_ = 0.371, *T*_max_ = 0.540	*l* = −13→13
3079 measured reflections	2 standard reflections every 120 min
2474 independent reflections	intensity decay: 1.3%

### Refinement

**Table:**

Refinement on *F*^2^	Primary atom site location: structure-invariant direct methods
Least-squares matrix: full	Secondary atom site location: difference Fourier map
*R*[*F*^2^ > 2σ(*F*^2^)] = 0.030	*w* = 1/[σ^2^(*F*_o_^2^) + (0.0429*P*)^2^ + 1.7705*P*] where *P* = (*F*_o_^2^ + 2*F*_c_^2^)/3
*wR*(*F*^2^) = 0.083	(Δ/σ)_max_ < 0.001
*S* = 1.09	Δρ_max_ = 1.52 e Å^−^^3^
2474 reflections	Δρ_min_ = −1.24 e Å^−^^3^
180 parameters	Extinction correction: *SHELXL97* (Sheldrick, 2008), Fc^*^=kFc[1+0.001xFc^2^λ^3^/sin(2θ)]^-1/4^
0 restraints	Extinction coefficient: 0.0295 (11)

### Special details

**Table:**

Geometry. All e.s.d.'s (except the e.s.d. in the dihedral angle between two l.s. planes) are estimated using the full covariance matrix. The cell e.s.d.'s are taken into account individually in the estimation of e.s.d.'s in distances, angles and torsion angles; correlations between e.s.d.'s in cell parameters are only used when they are defined by crystal symmetry. An approximate (isotropic) treatment of cell e.s.d.'s is used for estimating e.s.d.'s involving l.s. planes.
Refinement. Refinement of *F*^2^ against ALL reflections. The weighted *R*-factor *wR* and goodness of fit *S* are based on *F*^2^, conventional *R*-factors *R* are based on *F*, with *F* set to zero for negative *F*^2^. The threshold expression of *F*^2^ > σ(*F*^2^) is used only for calculating *R*-factors(gt) *etc*. and is not relevant to the choice of reflections for refinement. *R*-factors based on *F*^2^ are statistically about twice as large as those based on *F*, and *R*-factors based on ALL data will be even larger.

### Fractional atomic coordinates and isotropic or equivalent isotropic displacement parameters (Å^2^)

**Table:**

	*x*	*y*	*z*	*U*_iso_*/*U*_eq_	Occ. (<1)
Mo1	0.89052 (6)	0.41054 (5)	0.66707 (4)	0.01200 (14)	
Mo2	0.21772 (6)	0.16273 (5)	0.26393 (4)	0.01185 (14)	
Mo3	0.59566 (6)	0.76438 (5)	0.12859 (4)	0.01514 (14)	
Mn1	0.37898 (12)	0.45559 (10)	0.60871 (8)	0.01386 (18)	
Mn2	0.04434 (12)	0.70486 (9)	0.01860 (8)	0.01258 (18)	
Na3	0.2869 (3)	0.7921 (2)	0.80026 (18)	0.0198 (5)	0.829 (5)
Mn3	0.2869 (3)	0.7921 (2)	0.80026 (18)	0.0198 (5)	0.171 (5)
Na1	0.0000	0.0000	0.5000	0.0534 (13)	
Na2	0.5195 (17)	0.9729 (14)	0.4581 (10)	0.054 (4)	0.341 (9)
O1	0.7228 (9)	0.9722 (6)	0.2412 (6)	0.0554 (16)	
O2	0.8393 (8)	0.1999 (5)	0.5790 (5)	0.0320 (10)	
O3	0.7536 (7)	0.6998 (6)	0.0365 (5)	0.0320 (10)	
O4	0.2232 (6)	0.2013 (5)	0.4419 (4)	0.0205 (8)	
O5	0.1747 (7)	0.9506 (5)	0.1815 (5)	0.0282 (9)	
O6	0.1209 (6)	0.5181 (5)	0.6519 (4)	0.0206 (8)	
O7	0.0200 (6)	0.2252 (5)	0.1661 (4)	0.0213 (8)	
O8	0.3534 (6)	0.7437 (6)	0.0055 (4)	0.0247 (9)	
O9	0.6776 (5)	0.4713 (5)	0.5818 (4)	0.0169 (7)	
O10	0.9224 (6)	0.4447 (5)	0.8498 (4)	0.0169 (7)	
O11	0.4624 (6)	0.2725 (5)	0.2679 (4)	0.0197 (8)	
O12	0.5617 (8)	0.6484 (7)	0.2375 (5)	0.0443 (13)	

### Atomic displacement parameters (Å^2^)

**Table:**

	*U*^11^	*U*^22^	*U*^33^	*U*^12^	*U*^13^	*U*^23^
Mo1	0.0137 (2)	0.0142 (2)	0.0126 (2)	0.00713 (17)	0.00657 (16)	0.00746 (17)
Mo2	0.0132 (2)	0.0107 (2)	0.0131 (2)	0.00356 (16)	0.00512 (16)	0.00613 (16)
Mo3	0.0143 (2)	0.0171 (2)	0.0151 (2)	0.00401 (17)	0.00602 (17)	0.00724 (17)
Mn1	0.0142 (4)	0.0157 (4)	0.0155 (4)	0.0055 (3)	0.0078 (3)	0.0083 (3)
Mn2	0.0154 (4)	0.0126 (4)	0.0138 (4)	0.0062 (3)	0.0072 (3)	0.0073 (3)
Na3	0.0175 (9)	0.0210 (9)	0.0236 (9)	0.0081 (7)	0.0113 (7)	0.0070 (7)
Mn3	0.0175 (9)	0.0210 (9)	0.0236 (9)	0.0081 (7)	0.0113 (7)	0.0070 (7)
Na1	0.100 (4)	0.0235 (18)	0.080 (3)	0.031 (2)	0.076 (3)	0.031 (2)
Na2	0.032 (5)	0.046 (7)	0.061 (8)	−0.012 (4)	−0.015 (5)	0.032 (6)
O1	0.049 (3)	0.028 (3)	0.055 (3)	−0.010 (2)	0.016 (3)	−0.013 (2)
O2	0.050 (3)	0.018 (2)	0.031 (2)	0.016 (2)	0.017 (2)	0.0072 (18)
O3	0.021 (2)	0.046 (3)	0.034 (2)	0.016 (2)	0.0123 (18)	0.014 (2)
O4	0.026 (2)	0.0176 (18)	0.0165 (17)	0.0023 (16)	0.0092 (16)	0.0071 (15)
O5	0.037 (2)	0.0133 (18)	0.031 (2)	0.0050 (17)	0.0146 (19)	0.0034 (16)
O6	0.0193 (19)	0.027 (2)	0.0203 (19)	0.0108 (16)	0.0111 (16)	0.0099 (16)
O7	0.0162 (18)	0.030 (2)	0.024 (2)	0.0058 (16)	0.0070 (16)	0.0189 (17)
O8	0.020 (2)	0.037 (2)	0.030 (2)	0.0175 (18)	0.0116 (17)	0.0197 (19)
O9	0.0131 (17)	0.0257 (19)	0.0167 (17)	0.0078 (15)	0.0070 (14)	0.0116 (15)
O10	0.0221 (19)	0.0181 (18)	0.0162 (17)	0.0076 (15)	0.0091 (15)	0.0116 (15)
O11	0.0185 (18)	0.0196 (18)	0.0232 (19)	0.0075 (15)	0.0088 (16)	0.0086 (15)
O12	0.037 (3)	0.067 (4)	0.036 (3)	0.010 (3)	0.006 (2)	0.040 (3)

### Geometric parameters (Å, º)

**Table:**

Mo1—O2	1.726 (4)	Mn2—O7^v^	2.153 (4)
Mo1—O6^i^	1.772 (4)	Mn2—O10^iii^	2.161 (4)
Mo1—O10	1.787 (4)	Mn2—O8	2.194 (4)
Mo1—O9	1.812 (4)	Mn2—O10^vi^	2.264 (4)
Mo2—O5^ii^	1.739 (4)	Na3—O11^iii^	2.212 (4)
Mo2—O11	1.777 (4)	Na3—O1^vii^	2.250 (7)
Mo2—O4	1.778 (4)	Na3—O8^viii^	2.250 (4)
Mo2—O7	1.795 (4)	Na3—O7^ix^	2.279 (4)
Mo3—O1	1.742 (5)	Na3—O6	2.296 (4)
Mo3—O3	1.747 (4)	Na1—O2^iv^	2.376 (4)
Mo3—O12	1.757 (5)	Na1—O2^x^	2.376 (4)
Mo3—O8	1.790 (4)	Na1—O4^xi^	2.430 (4)
Mn1—O12^iii^	2.060 (4)	Na1—O4	2.430 (4)
Mn1—O6	2.148 (4)	Na1—O1^iii^	2.797 (6)
Mn1—O9	2.193 (4)	Na1—O1^xii^	2.797 (6)
Mn1—O9^iii^	2.224 (4)	Na2—O2^xiii^	2.408 (11)
Mn1—O4	2.224 (4)	Na2—O2^iii^	2.529 (12)
Mn1—O11^iii^	2.226 (4)	Na2—O4^iii^	2.815 (13)
Mn2—O3^iv^	2.115 (4)	Na2—O1	2.990 (12)
Mn2—O5	2.151 (4)		
			
O2—Mo1—O6^i^	107.4 (2)	O7^v^—Mn2—O8	83.66 (15)
O2—Mo1—O10	108.02 (19)	O10^iii^—Mn2—O8	95.72 (15)
O6^i^—Mo1—O10	110.69 (18)	O3^iv^—Mn2—O10^vi^	93.72 (16)
O2—Mo1—O9	108.4 (2)	O5—Mn2—O10^vi^	177.23 (16)
O6^i^—Mo1—O9	110.42 (17)	O7^v^—Mn2—O10^vi^	82.20 (14)
O10—Mo1—O9	111.77 (17)	O10^iii^—Mn2—O10^vi^	79.09 (14)
O5^ii^—Mo2—O11	107.62 (19)	O8—Mn2—O10^vi^	92.25 (15)
O5^ii^—Mo2—O4	108.4 (2)	O11^iii^—Na3—O1^vii^	104.9 (2)
O11—Mo2—O4	109.13 (18)	O11^iii^—Na3—O8^viii^	100.10 (15)
O5^ii^—Mo2—O7	110.2 (2)	O1^vii^—Na3—O8^viii^	130.52 (19)
O11—Mo2—O7	110.29 (17)	O11^iii^—Na3—O7^ix^	162.72 (17)
O4—Mo2—O7	111.16 (19)	O1^vii^—Na3—O7^ix^	87.64 (19)
O1—Mo3—O3	107.7 (3)	O8^viii^—Na3—O7^ix^	79.60 (15)
O1—Mo3—O12	107.1 (3)	O11^iii^—Na3—O6	79.28 (15)
O3—Mo3—O12	110.1 (3)	O1^vii^—Na3—O6	129.81 (19)
O1—Mo3—O8	110.5 (3)	O8^viii^—Na3—O6	96.19 (16)
O3—Mo3—O8	110.2 (2)	O7^ix^—Na3—O6	83.56 (15)
O12—Mo3—O8	111.1 (2)	O2^iv^—Na1—O2^x^	180.000 (1)
O12^iii^—Mn1—O6	94.94 (19)	O2^iv^—Na1—O4^xi^	90.01 (15)
O12^iii^—Mn1—O9	93.48 (18)	O2^x^—Na1—O4^xi^	89.99 (15)
O6—Mn1—O9	163.16 (15)	O2^iv^—Na1—O4	89.99 (15)
O12^iii^—Mn1—O9^iii^	169.9 (2)	O2^x^—Na1—O4	90.01 (15)
O6—Mn1—O9^iii^	92.19 (14)	O4^xi^—Na1—O4	180.0
O9—Mn1—O9^iii^	81.49 (14)	O2^iv^—Na1—O1^iii^	101.44 (16)
O12^iii^—Mn1—O4	89.8 (2)	O2^x^—Na1—O1^iii^	78.56 (16)
O6—Mn1—O4	101.04 (16)	O4^xi^—Na1—O1^iii^	80.94 (15)
O9—Mn1—O4	93.54 (15)	O4—Na1—O1^iii^	99.06 (15)
O9^iii^—Mn1—O4	81.83 (14)	O2^iv^—Na1—O1^xii^	78.56 (16)
O12^iii^—Mn1—O11^iii^	103.0 (2)	O2^x^—Na1—O1^xii^	101.44 (16)
O6—Mn1—O11^iii^	82.21 (15)	O4^xi^—Na1—O1^xii^	99.06 (15)
O9—Mn1—O11^iii^	81.69 (14)	O4—Na1—O1^xii^	80.94 (15)
O9^iii^—Mn1—O11^iii^	84.99 (14)	O1^iii^—Na1—O1^xii^	180.0
O4—Mn1—O11^iii^	166.52 (14)	O2^xiii^—Na2—O2^iii^	156.6 (5)
O3^iv^—Mn2—O5	88.82 (18)	Na2^vii^—Na2—O4^iii^	104.7 (13)
O3^iv^—Mn2—O7^v^	93.21 (17)	O2^xiii^—Na2—O4^iii^	80.8 (3)
O5—Mn2—O7^v^	98.77 (16)	O2^iii^—Na2—O4^iii^	104.7 (4)
O3^iv^—Mn2—O10^iii^	89.32 (17)	Na2^vii^—Na2—O1	154.6 (14)
O5—Mn2—O10^iii^	99.87 (16)	O2^xiii^—Na2—O1	74.3 (3)
O7^v^—Mn2—O10^iii^	161.24 (15)	O2^iii^—Na2—O1	128.2 (4)
O3^iv^—Mn2—O8	172.82 (18)	O4^iii^—Na2—O1	86.6 (4)
O5—Mn2—O8	85.29 (17)		

Symmetry codes: (i) *x*+1, *y*, *z*; (ii) *x*, *y*−1, *z*; (iii) −*x*+1, −*y*+1, −*z*+1; (iv) *x*−1, *y*, *z*; (v) −*x*, −*y*+1, −*z*; (vi) *x*−1, *y*, *z*−1; (vii) −*x*+1, −*y*+2, −*z*+1; (viii) *x*, *y*, *z*+1; (ix) −*x*, −*y*+1, −*z*+1; (x) −*x*+1, −*y*, −*z*+1; (xi) −*x*, −*y*, −*z*+1; (xii) *x*−1, *y*−1, *z*; (xiii) *x*, *y*+1, *z*.
